# Antioxidant Activities of *Caragana sinica* Flower Extracts and Their Main Chemical Constituents

**DOI:** 10.3390/molecules15106722

**Published:** 2010-09-28

**Authors:** Zhi-Gang Tai, Le Cai, Lin Dai, Wei-Jun Sun, Wei Zhe, Ya-Bin Yang, Qiu-E Cao, Zhong-Tao Ding

**Affiliations:** 1 Key Laboratory of Medicinal Chemistry for Nature Resource, Ministry of Education, School of Chemical Science and Technology, Yunnan University, Kunming 650091, Yunnan, P.R. China; E-Mails: tzgzj@163.com (Z.G.T.); caile@ynu.edu.cn (L.C.); truesun18@hotmail.com (W.J.S.); ybyang@ynu.edu.cn (Y.B.Y.); qecao@ynu.edu.cn (Q.E.C.); 2 Department of Applied mathematics, Kunming University of Science and Technology, Kunming 650093, P.R. China; E-Mail: Dailin1968@sina.com.cn (L.D.); 3 Technology Center, Hongyun-Honghe Tobacco (Group) Co. Ltd., Kunming 650202, P.R. China; E-Mail: Zhewei11@163.com (W.Z.)

**Keywords:** *Caragana sinica*, antioxidant activity, flavonoid

## Abstract

The edible flowers of *Caragana sinica* are used in China a kind of health-promoting vegetable. In this study, the antioxidant activities of its ethanol extract, as well as its petroleum ether, ethyl acetate, *n*-butanol and water fractions, were evaluated through *in vitro* model systems including the DPPH, FRAP and β-carotene bleaching methods. Among the *C. sinica* flower extracts the ethyl acetate fraction was the most effective. Correlation analysis suggested that flavonoids might be the major contributors to the high antioxidant activity of this flower. Six flavonoids including quercetin (**1**), isoquercitrin (**2**), rutin (**3**), quercetin-3′-*O*-methyl-3-*O*-α-L-rhamnopyranosyl (1→6)-β-D-glucopyranoside (**4**), typhaneoside (**5**) and quercetin-3-*O*-β-D-glucopyranosyl(1→2)[α-L-rhamnopyranosyl (1→6)]-β-D-glucopyranoside (**6**), were isolated from this flower for the first time. Their contents were determined by a HPLC method. Compounds **3** and **4** were found to be the major flavonoids, with concentrations of 1.20 ± 0.07 and 3.94 ± 0.12 mg/g dry sample, respectively. These results demonstrated that the *C. sinica* flowers may be valuable natural antioxidant sources and are potentially applicable in the health food industry.

## 1. Introduction

It is well known that free radicals can cause molecular transformations and gene mutations in many kinds of organisms. In the healthy body, free radicals are continuously balanced by the natural antioxidant defense system. Once the balance is destroyed, nucleic acids, lipids and proteins can suffer oxidative damage, resulting in tissue injury [1]. Although organisms have endogenous antioxidants which can keep the balance between antioxidants and oxidants in living organisms in some extent, increased intakes of dietary antioxidants may be important to inhibit the oxidation of an oxidizable substrate in organisms in a chain reaction [2]. On the other hand, antioxidants are widely used as food additives to provide protection against oxidative degradation of foods by free radicals [3]. Therefore, it is very significant to find antioxidants from natural resources. Various plant extracts have exhibited good antioxidant properties [4,5,6,7,8].

*Caragana sinica* (Buc’hoz) Rehd. is widely distributed in China. Its yellow flowers are solitary, axillary. As a kind of vegetable, *C .* *sinica* flowers is used in cooking eggs, meats, and soups as a remedy for dehydration, indigestion, hypertension, dizziness, tinnitus and cough in some provinces of China, such as Jiangsu, Shandong, Hebei, Shanxi, Neimenggu, Ningxia, *etc.* Especially in Yunnan province of China, some minority races, such as the Bai, Yi, Miao, *etc.*, have the tradition of eating *C .* *sinica* flowers. It is said that eating the flower in springtime could eliminate the ‘toxin’ which had been accumulated in the human body in the previous season [9].

**Figure 1:**
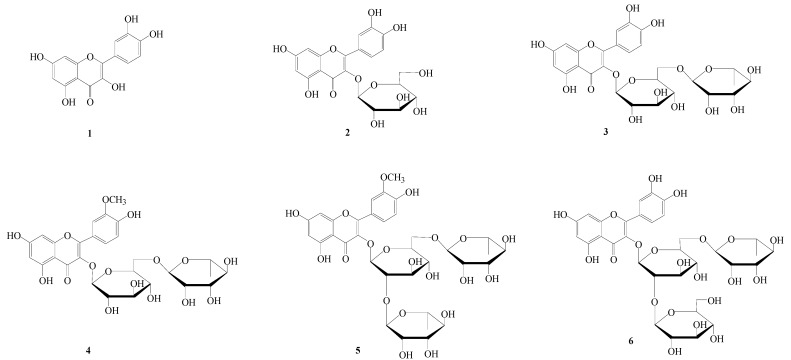
Chemical structures of compounds **1**–**6**.

Previous studies have focused on the chemical constituents and bioactivity capacity of *C. sinica* roots [10,11,12,13,14], but there is little study on the antioxidant activity and constituents of *C. sinica* flowers. In this paper, the antioxidant capacities of the ethanol extract and its derived soluble fractions from *C. sinica* flowers were evaluated by various *in vitro* methods including the DPPH (radical-scavenging activity), FRAP (ferric reducing activity) and β-carotene bleaching assays. The contents of total phenolics and total flavonoids were determined. Moreover, further investigation on the constituents of this flower led to the isolation of six flavonoids for the first time, namely quercetin (**1**), isoquercitrin (**2**), rutin (**3**), quercetin-3′-*O*-methyl-3-*O*-α-L-rhamnopyranosyl-(1→6)-β-D-glucopyranoside (**4**), typhaneoside (**5**) and quercetin-3-*O*-β-D-glucopyranosyl(1→2)[α-L-rhamnopyranosyl (1→6)]-β-D-glucopyranoside (**6**), respectively (Figure 1). Finally, the contents of these compounds were determined by a HPLC method.

## 2. Results and Discussion

### 2.1. Antioxidant Activities

**Figure 2:**
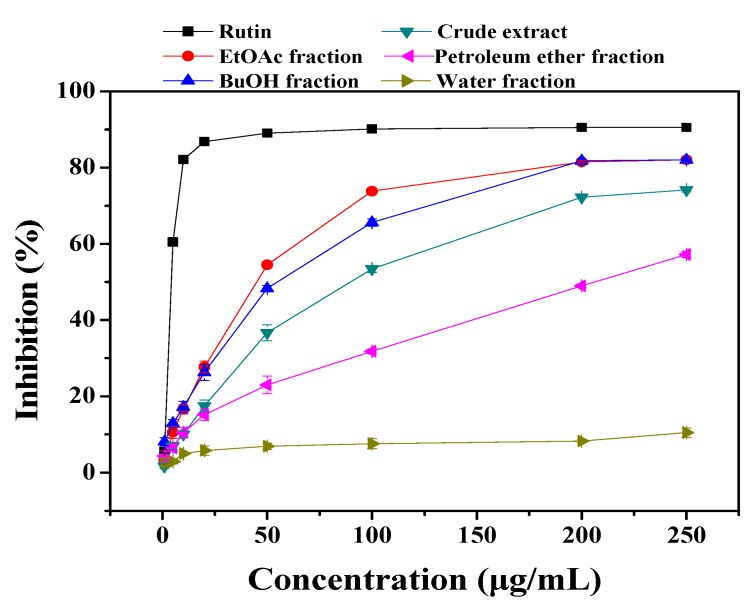
DPPH radical-scavenging activities of ethanol extract and its derived fractions of *C . sinica* flowers. Values were means ± SD (*n* = 3). P < 0.05.

Three methods including DPPH radical scavenging activity, ferric reducing/antioxidant power (FRAP), and β-carotene bleaching assays were used to evaluate antioxidant activities of the ethanol extract and its derived soluble fractions of *C. sinica* flowers. DPPH• is a stable free radical with a characteristic absorption at 517 nm, which is widely used to study the radical-scavenging activity of natural antioxidants [15]. The results of DPPH assay were shown in Figure 2.

Except for the water fraction, all the other fractions and the crude ethanol extract showed DPPH radical scavenging activity. The activity of the EtOAc fraction (IC_50_ = 45.0 μg/mL) was the highest, followed by the BuOH fraction (IC_50_ = 55.1 μg/mL), crude extract (IC_50_ = 90.1 μg/mL), and petroleum ether fraction (IC_50_ = 206.1 μg/mL), respectively. Compared with the extracts of dill flower (*Anethum graveolens* L), for which a DPPH radical scavenging effect with an IC_50_ value of 85.3 μg/mL was reported [5], the EtOAc and BuOH fractions of the *C. sinica* flowers were more effective in scavenging DPPH radicals*.*

Generally, reducing properties are associated with the presence of compounds which exert their action by breaking the free radical chains by donating a hydrogen atom [16]. The FRAP assay was used for the analysis of the antioxidant activities of *C. sinica* flower extracts. The results of the FRAP assay were consistent with those of the DPPH radical-scavenging test. The EtOAc fraction showed the highest FRAP value of 2557 ± 31 μmol Fe^2^^+^/g dry extract, followed by the BuOH fraction (1582 ± 22 μmol Fe^2^^+^/g dry extract), crude extract (659 ± 29 μmol Fe^2^^+^/g dry extract), petroleum ether fraction (348 ± 17 μmol Fe^2^^+^/g dry extract), and water fraction (28 ± 4 μmol Fe^2^^+^/g dry extract). This implied that EtOAc fraction had stronger reducing power than the other fractions.

The presence of antioxidants can hinder the extent of β-carotene bleaching by acting on the hydroperoxides formed in the system [17]. The degradation rate of β-carotene-linoleate depends on the antioxidant activity of the extracts, and the extracts with the lowest β-carotene degradation rate exhibited the highest antioxidant activity. As shown in Table 1, the oxidation rate ratio (R_OR_) of the crude extract and its derived soluble fractions from the flowers of *C. sinica* were 0.52 ± 0.005 (crude extract), 0.58 ± 0.006 (petroleum ether fraction), 0.26 ± 0.004 (EtOAc fraction), 0.41 ± 0.005 (BuOH fraction) and 0.51 ± 0.005 (water fraction). Their antioxidant activities (AA%) followed the order of EtOAc fraction>BuOH fraction>crude extract ≈ water fraction> petroleum ether fraction.

**Table 1:** Antioxidant activities of crude extract and its derived fractions of *C. sinica* flowers assayed by β-carotene bleaching method. Final concentration was 5 μg/mL. All the AA% and R_OR_ values were calculated at t = 120 min. Values of each data were means ± SD (*n* = 3). P < 0.05.

Sample	Antioxidant activity (AA%)	Oxidation rate ratio (R_OR_)
EtOAc fraction	73.57 ± 0.63	0.26 ± 0.004
BuOH fraction	59.12 ± 0.56	0.41 ± 0.005
Crude extract	47.85 ± 0.53	0.52 ± 0.005
Petroleum ether fraction	42.11 ± 0.46	0.58 ± 0.006
Water fraction	48.77 ± 0.49	0.51 ± 0.005

### 2.2. Contents of Total Phenolics and Total Flavonoids

Phenolic compounds such as flavonoids, phenolic acids, and tannins are considered to be major contributors to the antioxidant capacities of plants [4]. Table 2 showed the values of total phenolic content (TPC) in the crude extract and its derived fractions of *C. sinica* flowers, expressed in terms of mg gallic acid/g dry extract. The results revealed that the TPC of the EtOAc fraction (140.0 ± 10.1 mg gallic acid/g dry extract) was much higher than that of other fractions. The TPC values increased in the following order: water fraction, petroleum ether fraction, crude extract, BuOH fraction, and EtOAc fraction. Table 2 also shows the total flavonoid contents of the crude extract and its derived fractions. The results were expressed in mg rutin/g dry extract by comparison with standard rutin treated in the same conditions. The results revealed that the level of flavonoids in *C. sinica* flowers was considerable, and total flavonoid contents of different fractions decreased in the same order of TPC. The EtOAc fraction (157.8 ± 11.4 mg rutin/g dry extract) contained more total flavonoids than all other fractions. Some investigations also indicated that the considerable amounts of phenols, and flavonoids existed in some flowers, such as chestnut flower and lychee flower [18,19].

**Table 2:** Total phenolic contents and total flavonoids contents of crude extract and its derived soluble fractions of *C . sinica* flowers. Values of each data were means ± SD (*n* = 3). P < 0.05.

Sample	Total phenolic content (TPC)(mg gallic acid /g dry extract)	Total flavonoid content (TFC) (mg rutin /g dry extract)
EtOAc fraction	140.0 ± 10.1	157.8± 11.4
BuOH fraction	117.5 ± 8.4	100.6 ± 6.8
Crude extract	68.0 ± 4.7	69.9 ± 3.4
Petroleum ether fraction	31.7 ± 1.8	15.5 ± 2.3
Water fraction	7.9 ± 0.7	4.9 ± 0.5

### 2.3. Correlation Analysis

Correlation analysis between total antioxidant content and antioxidant activity was carried out (Table 3).

**Table 3:** Correlations established between total phenolics and flavonoids with antioxidant activities.

Assay	Equation			
	Total phenolic content (TPC)	*R^2^*	Total flavonoid content (TFC)	*R^2^*
Total flavonoids content	*y* = 1.1042*x* - 10.8870	0.9566	-	-
Frap assay	*y* = 17.8586*x* - 269.2349	0.9349	*y* = 15.9845*x* - 79.9588	0.9546
EC_50_ of DPPH radical-scavenging activity^ a^	*y* = -1.3945*x* + 223.6241^a^	0.8470^ a^	*y* = -1.1250*x* + 195.7892^ a^	0.8186^ a^
*β*-carotene bleaching inhibition	*y* = 0.1931*x* + 40.1867	0.7534	*y* = 0.1807*x* + 41.6832	0.8410

a Except for the water fraction, crude extract and its EtOAc, BuOH and petroleum ether fractions from *C. sinica* flowers were used in the correlation analysis.

TPC and TFC provided a strong correlation with the FRAP assay in this study (*R^2^* = 0.9349 and 0.9546, respectively). This result was in agreement with the reports by Kubola and Siriamornpun [20]. Similar results were also found for the DPPH radical scavenging activity (*R^2^* = 0.8470 and 0.8186). Moreover, TPC also exhibited good correlation with TFC (*R^2^* = 0.9566). This implied that the flavonoids were an important phenolic group in the *C. sinica* flower extract, and the higher TPC could be mostly attributed to the more abundant flavonoids in the *C. sinica* flowers.

Some papers have reported that there was no significant correlation between total phenolic content and β-carotene bleaching assay results [21]. However, in our study there was a noteworthy correlation (*R^2^* = 0.7534 or 0.8410) between TPC (or TFC) and the β-carotene bleaching assay. This supported the results of β-carotene bleaching assay reported by Shyu, *et al.* [5] for different fractions of dill flower (*R^2^* >0.95). On the other hand, all the results provided further evidence that the good antioxidant activities measured by these three chemical assays could be attributed to the flavonoids in the flowers of *C. sinica*.

### 2.4. Isolation and Determination of Flavonoid Compounds

In order to obtain the compounds, especially flavonoid compounds, which might be the main contributors to the high antioxidant activity of *C .* *sinica* flowers, an investigation on the chemical constituents of this flower was carried out. Six compounds were isolated and purified. On the basis of physicochemical properties, spectral data (MS, NMR and IR), the structures were identified to be quercetin (**1**), isoquercitrin (**2**), rutin (**3**), quercetin-3′-*O*-methyl-3-*O*-α-L-rhamnopyranosyl-(1→6)-β-D-gluco-pyranoside (**4**), typhaneoside (**5**) and quercetin-3-*O*-β-D-glucopyranosyl(1→2)[α-L-rhamnopyranosyl (1→6)]-β-D-glucopyranoside (**6**).

**Figure 3:**
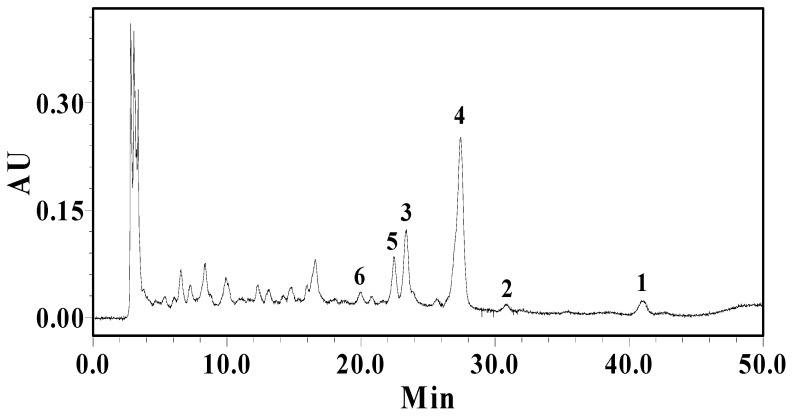
HPLC analysis of the ethanol extract of *C. sinica* flowers.

The contents of compounds **1**-**6** were determined by HPLC analysis (Figure 3). The results revealed that compounds **4** (3.94 ± 0.12 mg /g dry sample) and **3** (1.20 ± 0.07 mg /g dry sample) were the major compounds in *C .* *sinica*flowers, followed by compound **5** (0.95 ± 0.07 mg /g dry sample), compound **1** (0.45 ± 0.05 mg /g dry sample), compound **6** (0.36 ± 0.03 mg /g dry sample), and compound **2** (0.29 ± 0.04 mg /g dry sample), respectively.

Quercetin (**1**) and rutin (**3**) have been demonstrated to possess significant antioxidant activity because of the presence of the 3′,4′-orthodihydroxy configuration in their ring B of chemical structures [22,23]. Both compounds **2** and **6** have the same aglycone (quercetin) as rutin, so it could be inferred that **2** and **6** should exhibit the high antioxidant activity, too. Therefore, it may be concluded that the antioxidant activities of *C. sinica* flowers is most probably a synergistic effect of compounds **1**–**3**, and **6**. On the other hand, the highest concentration of **4** in the extract of *C. sinica* flowers was taken into account, **4** with lower antioxidant activity than compounds **1**–**3**, and **6**, may also contribute to the antioxidant activities of *C. sinica* flowers in some extent.

## 3. Experimental

### 3.1. Plant Material, Chemicals and Equipment

The *Caragana sinica* flowers were collected in Xishan County, Kunming, Yunnan Province, P.R. China, in April 2008, and authenticated by Prof. Shugang Lu (Yunnan University, Kunming, P.R. China). A voucher specimen (2008-Ding-Tai-2) was deposited in the School of Chemical Science and Technology, Yunnan University.

Silica gel (200-300 mesh) and silica gel GF_254_ plates were purchased from Qingdao Marine Chemical Co. (Qingdao, P.R. China). Sephadex LH-20 and Rp18 were purchased from Merck (Darmstadt, Germany). 2,2'-Diphenyl-1-picrylhydrazyl (DPPH), 2,4,6-tri-(2-pyridyl)-*s*-triazine (TPTZ), Folin-Ciocalteu reagent, gallic acid and rutin were purchased from Sigma (St. Louis, MO, USA). Methanol (HPLC grade) was purchased from Fisher Chemicals (New Jersey, USA). Water used was purified with a labpure water system (Aike, Chengdu, China). All other reagents from local sources were of analytical grade.

A Shimadzu UV-VIS-PS 2410 spectrometer (Tokyo, Japan) was used for colorimetric measurements. The HPLC system consisted of a Waters 1525 pump and a Waters 2996 PAD detector (Waters Corporation, Milford, MA, USA). The column was XTerra-C_18_ reversed-phase column (250 × 4.6 mm, 5 μm; Waters). MS were measured on an Agilent G3250AA LC/MSD TOF spectrometer. NMR spectra were recorded on a Bruker DRX-500 NMR spectrometer (Kleve, Germany) using Me_4_Si as an internal standard.

### 3.2. Extraction and Isolation of Compounds.

Powdered dry flowers of *C . sinica* (4.5 kg) were extracted three times with 85% ethanol (20 L, each 48 h) at room temperature. After the solvent was removed under reduced pressure at 40 °C, the ethanol extract (565.5 g) was suspended in 3 L of water and partitioned with 3 L of petroleum ether, 3 L of ethyl acetate (EtOAc), 3 L of *n*-butanol (BuOH) to yield the petroleum ether (145.6 g), EtOAc (101.0 g), BuOH (136.7 g) and water (138.3 g) fractions, respectively. A sample (5 g) was scooped out from each fraction for antioxidant experiments. The rest was used to study their chemical constituents.

The EtOAc fraction was subjected to silica gel column chromatography (chloroform-methanol = 1:0→0:1) to obtain six fractions (Fr_E_.1-Fr_E_.6) on the basis of TLC analysis. Fr_E_.4 (25.5g) was subjected to a Sephadex LH-20 column (acetone) and further purified on a silica gel column (petroleum ether-acetone = 4:1→1:1; petroleum ether-ethyl acetate = 2:1) to yield compound **1** (26 mg) and compound **2** (37 mg). The BuOH fraction was subjected to silica gel column chromatography (chloroform-methanol = 1:0→ 0:1), to give seven fractions (Fr_B_.1-Fr_B_.7). compound **4** (1.5 g ) was obtained by crystallization from the Fr_B_.4. Fr_B_.5 (8.0 g) was chromatographed on a Sephadex LH-20 column (methanol) and further purified by silica gel column chromatography (ethyl acetate -methanol = 3:1) to give compound **3** (17 mg). Fr_B_. 6 (6.4 g) was separated by silica gel column (ethyl acetate- methanol = 10:1→0:1), RP_18_ (methanol-water = 1:2→2:1) and Sephadex LH-20 (methanol) to yield compounds **5** (23 mg) and **6** (15 mg), respectively. The structures of compounds **1-6** (Figure 1) were elucidated by NMR, MS analysis and comparison with previously reported data.

### 3.3. Determination of Antioxidant Activity

*DPPH radical-scavenging* *assay:* The DPPH free radical scavenging capability was performed as described by Hung *et al.* [24]. DPPH (3.9 mL, 0.075 mM) was mixed with properly diluted sample (100 μL). After storage at room temperature for 30 min, the absorption of the reaction mixture was recorded at 517 nm against a blank. Inhibition of DPPH radical scavenging activity in percent (I%) was calculated according to the equation of I% = [(A_blank_− A_sample_)/ A_blank_] × 100 where A_sample_ is the absorbance of the sample, and A_blank_ is the absorbance of blank solution (containing all reagents except the test sample).

*Ferric reducing activity based on FRAP assay:* Ferric reducing antioxidant power was measured according to the method described by Suárez *et al.* with some modification [25]. The working FRAP reagent was prepared with TPTZ (5 mL, 10 mM), FeCl_3_ (5 mL, 20 mM) and sodium acetate buffer (50 mL, 300 mM, pH = 3.6). The freshly prepared FRAP reagent (3.0 mL) was mixed with sample solution (100 μL) and deionized water (300 μL). The reaction mixture was incubated for 30 min at 37 °C in a water bath. The absorbance was recorded at 595 nm. A standard solution of Fe^2+^ was used to prepare a calibration curve. In this assay, all solutions were used on the day of preparation.

*β-carotene bleaching method:* The inhibition activity of β-carotene oxidation by peroxide radicals of the samples was determined according to a modified method, which described by Wu *et al.* [26]. β-carotene (2.0 mg) was dissolved in chloroform (10.0 mL). This β-carotene solution (1.0 mL) was pipetted into a measuring flask (100 mL) containing linoleic acid (20 mg) and Tween 40 (200 mg). The chloroform was removed at 30 °C for 10 min using a rotary evaporator. After evaporation, distilled water (100 mL) was added to the mixture with vigorous agitation to form an emulsion. Two mL of this emulsion and sample solution (100 μL) were transferred into a test tube. The mixture was then gently mixed and placed in a water bath at 50 °C for 2 h. Absorbance of the solution was recorded every 20 min up to 120 min at 470 nm. Blank sample consisted of solvent (0.2 mL) instead of sample solution. The results were based upon two different parameters of the antioxidant activity (AA%) and the oxidant rate ratio (R_OR_). The antioxidant activity (AA%) and oxidant rate ratio (R_OR_) were calculated according to the following equation from Kubola and Siriamornpun [18]: AA% = [R _blank_− R _sample_ /R _blank_] × 100, where *R*_sample_ and *R*_blank_ represent the bleaching rates of β-carotene with or without the addition of sample, respectively; Degradation rates (R_D_) were calculated according to following equation: R_D_ = ln (A _t=0_ / A _t=x_) × 1/t, where A _t=0_ is the absorbance at 470 nm at t = 0 and A _t=x_ is the absorbance at 470 nm at t = 20, 40, 60, 80, 100, 120 min, respectively; The oxidation rate ratio (R_OR_) was calculated by the equation of R_OR_ =(R_sample_ /R_blank_).

### 3.4. Determination of Total Antioxidant Content

*Total phenolic content:* The total phenolic content was determined according to the Folin-Ciocalteau method [27] with minor modifications. Properly diluted sample (100 μL) was mixed with Folin-Ciocalteau reagent (4.5 mL) which was pre-diluted 10 times with deionized water. After standing for 5 min at room temperature, NaCO_3_ (3.0 mL, 7.5%, w/v) was added, and this mixture solution was incubated at room temperature for 60 min. The absorbance was measured at 765 nm. A standard solution of gallic acid was used to prepare a calibration curve. The total phenolic content (TPC) was calculated and expressed as mg gallic acid /g of dry extract.

*Total* *flavonoids content:* Total flavonoids content was determined by a colorimetric assay based on the method employed by Siddhuraju and Becker with slight modifications [28]. The properly diluted sample (1.0 mL) was mixed with deionized water (4.0 mL) in a 10.0 mL volumetric flask, and NaNO_2_ (0.3 mL, 5.0%, w/v) was added. After 5 min, AlCl_3_ (0.3 mL, 10%, w/v) was added. Then, after 6 min, NaOH (2.0 mL, 1.0 M) was added to the mixture, and followed by the addition of deionized water (2.4 mL). The solution was mixed and shaken vigorously, and the absorbance at 510 nm was measured against a blank. A standard solution of rutin was used to prepare a calibration curve. The results were expressed as mg rutin /g of dry extract.

### 3.5. HPLC Analysis

The air-dried and powdered flowers of *C. sinica* (1.0 g) were extracted three times each with 85% aqueous ethanol (20 mL) at room temperature. The resulting crude extract of the *C.sinica* flowers and authentic samples were prepared as solutions (10.00 mg/mL and 1.00 mg/mL, respectively). All the solutions were filtered through a 0.45 μm filter before injection into the HPLC. The optimal mobile phase for analysis was a gradient elution system consisting of solvent A (water, 5.0% acetic acid) and solvent B (methanol). The gradient program was as follows: 0-10 min, 10-30% solvent B; 10-20 min, 30-40% solvent B; 20-40 min, 40-43% solvent B; 40-50 min, 43-80%. The flow rate was 1.0 mL/min, and the column temperature was set at 40 °C. The injection volume was 20 μL. The UV detection wavelength was monitored at 280 nm. The peaks were confirmed by the UV absorptions and retention times of authentic sample.

### 3.6. Statistical Analysis

All samples were tested and analyzed in triplicate. Results were calculated as the mean ± SD (standard deviation) for each sample. Statistical analysis was done with one way analysis of variance. The correlation coefficient (*R^2^*) was used to show correlations. A significant difference was judged to exist at a level of *P* < 0.05.

## 4. Conclusions

The present study indicated that *C. sinica* flowers are rich in flavonoids and exhibit strong antioxidant activity in the three methods tested (DPPH, FRAP and β-carotene bleaching assays). Its EtOAc soluble fraction showed the strongest antioxidant activity. The antioxidant activities correlated well with their content of flavonoid compounds. Although the antioxidant activity found in an *in vitro* experiment is only indicative for the potential health benefits, these results remain significant as the first step in screening antioxidant activity of *C. sinica* flowers. It can be concluded that, *C. sinica* flowers, which are consumed as a vegetable in China, can be used as an accessible source of natural antioxidants with consequent health benefits.
